# What needs to change to increase chlamydia screening in general practice in Australia? The views of general practitioners

**DOI:** 10.1186/1471-2458-8-425

**Published:** 2008-12-30

**Authors:** Jane S Hocking, Rhian M Parker, Natasha Pavlin, Christopher K Fairley, Jane M Gunn

**Affiliations:** 1 Key Centre for Women's Health in Society, School of Population Health, University of Melbourne, Melbourne, Australia; 2 Department of Health Science, Monash University, Melbourne and Healthpact Research Centre, University of Canberra, Canberra, Australia; 3 Department of General Practice, University of Melbourne, Melbourne, Australia; 4 School of Population Health, University of Melbourne and the Melbourne Sexual Health Centre, Melbourne, Australia

## Abstract

**Background:**

Australia is considering implementing a chlamydia screening program in general practice. The views of general practitioners (GPs) are necessary to inform the design of the program. This paper aimed to investigate Australian GPs' views on how chlamydia screening could work in the Australian context.

**Methods:**

This project used both qualitative interviews and a quantitative questionnaire. GPs were randomly selected from a national database of medical practitioners for both the qualitative and quantitative components. Semi-structured interviews were conducted with GPs and a thematic analysis conducted. The results of the interviews were used to design a quantitative postal questionnaire for completion by a larger sample of GPs. Up to three reminders were sent to non-responders.

**Results:**

Twenty one GPs completed an interview and 255 completed the postal questionnaire. The results of the postal survey were in strong concordance with those of the interview. GPs identified a number of barriers to increased screening including lack of time, knowledge of GPs and the public about chlamydia, patient embarrassment and support for partner notification. GPs felt strongly that screening would be easier if there was a national program and if the public and GPs had a greater knowledge about chlamydia. Incentive payments and mechanisms for recall and reminders would facilitate screening. Greater support for contact tracing would be important if screening is to increase.

**Conclusion:**

Chlamydia screening in general practice is acceptable to Australian GPs. If screening is to succeed, policy makers must consider the facilitators identified by GPs.

## Background

*Chlamydia trachomatis *infection is the most commonly notified sexually transmissible infection (STI) in Australia, with over 50,000 infections notified in 2007 (244 per 100,000 population) [1]. Australian data show the greatest burden of chlamydia infection is among young women aged 15 to 24 years with a prevalence of about 3–4% among this age group [2,3]. Infection with chlamydia can have serious consequences, particularly for women. Up to two thirds of cases of tubal infertility and one third of cases of ectopic pregnancy are potentially attributable to past chlamydia infection [4].

Many countries around the world already have screening programs in place including Sweden [5] and the United Kingdom [6] and others including the Netherlands [7], Denmark [7] and Australia [8] are still considering how best to implement screening.

In its first National Sexually Transmissible Infections Strategy released in 2005 [9], the Australian Commonwealth Government stated that a chlamydia screening pilot program targeting sexually active young people under 25 years of age should be a priority action. In response, it announced in 2007 that it would evaluate a chlamydia screening pilot program set in general practice; the design of this program is currently under development [10]. Unlike general practice in the United Kingdom, people in Australia are not required to register with a single clinic, but can attend several different clinics. However, general practitioners (GPs) in Australia are ideally placed to conduct widespread chlamydia screening as nearly 90% of Australian women aged 15–24 years of age, the key risk group,[4] visit a GP at least once each year [11], and the many specialist youth, family planning and STI clinics and outreach screening programs targeting the homeless and marginalized populations help to reach the remaining 10% [12-14].

Successful programs require support from both the professionals who will undertake the screening and the target group to be screened [15]. As GPs are likely to be conducting the vast majority of chlamydia tests, it is vital that their opinions are sought before planning a chlamydia screening program in Australia. This study aimed to determine GPs' views on how chlamydia screening could work in the Australian context.

## Methods

This study included both qualitative and quantitative components and was conducted in Victoria (population 5 million), a State of Australia during 2005 and 2006. The results of the qualitative interviews were used to design a self-administered questionnaire that aimed to explore the findings of the interviews on a larger sample of GPs. We were careful to balance individual or discipline bias in assembling our research team: an experienced sexual health and infectious diseases physician, two practising GPs with academic experience, a sociologist with expertise in qualitative research and an epidemiologist with a particular interest in chlamydia infection.

### Qualitative – semi-structured interviews

A random selection of 1,000 GPs in Victoria (minimum sample size available for purchase) was acquired from the Australian Medical Publishing Company's (AMPCo) national database of medical practitioners [16]. This sample of 1000 was stratified by geographical location proportional to population size, based on GP postcode (rural versus metropolitan) and a random sample of 70 GP names selected proportionately across these strata, was taken to ensure that both rural and metropolitan GPs were invited to participate. They were considered eligible to be interviewed if they were currently practising as a GP in Victoria.

RP conducted all interviews face to face, following a semi-structured form. As annual screening for young women is currently recommended in Australia [17], the interviews focused on screening among young women, although we acknowledge the importance of including men in screening. Recruitment continued until saturation of emerging themes was evident during the interviews. The interview schedule was devised with input from all members of our multi-disciplinary research team and was informed by current knowledge and policy about chlamydia control. Interviews were transcribed verbatim and imported into NVivo7. We applied a thematic analysis and looked for emerging themes [18]. NP and RP read all transcripts, worked on the thematic analysis separately and met to discuss emerging themes. The coding framework developed from these discussions. NP and RP reviewed emerging themes as the analysis progressed. We attempted to avoid bias in interpreting the data by having both RP and NP active in reviewing the transcripts, eliciting themes and formulating our analysis.

### Quantitative – postal questionnaire

The postal questionnaire was pilot tested among a sample of eight medical practitioners known to the study investigators prior to administration. Assuming a response rate of 50%, a sample size of 300 would give 95% confidence intervals around a proportion of 50% from 44% to 56%. On this basis, a further random sample of 600 GPs was selected from the 1000 sample described above. GPs were sent a letter of invitation, a copy of the questionnaire and a reply paid envelope. Up to three reminders were sent to non-responding GPs. The postal questionnaire was analysed using Stata version 9 [19].

### Ethics

Ethics approval for this project was obtained from the University of Melbourne Human Research Ethics Committee.

## Results

In-depth interviews were conducted with 21 GPs (30% of those contacted) – 13 GPs interviewed were in urban practices and eight in rural and regional practices, eight were female. Of the 600 GPs selected to complete the postal questionnaire, 543 were confirmed to be still practicing in general practice and 255 participated with a response rate of 47%. Female GPs [45.9% (95%CI: 39.6%, 52.2%)] and older GPs [mean age 49.8 years (95%CI: 48.7 years, 50.9 years)] were more likely to complete the postal questionnaire compared with the whole Victorian GP population (35.4% female GPs, mean age = 47.7 years) [20]. The results of these two studies are presented together for each issue investigated.

### GPs' attitudes to screening

#### Qualitative component

There was overwhelming support for screening amongst those interviewed; GPs felt that general practice was an appropriate site for screening. Opportunistic screening for chlamydia during a sexual health consultation was the preferred model with most mentioning the Pap test (cervical smear) as an opportune time to conduct a chlamydia test. Other GPs mentioned screening when symptoms are present or during a sexual health consultation (other than the Pap test):

Yes, I think it should take place in general practice, because I think if we're aiming at young women ... ..... that's who they're going to come in contact with... on a first line basis. (DC13)

...* at the moment it's marketed with STD checks, which people often come and do. I think that's one way to do it, to offer with STD checks. (DC20)*

#### Quantitative component

The postal questionnaire revealed similar results to those obtained during the interviews. GPs believed chlamydia screening is a GP's responsibility: 90% of respondents disagreed with the statement "*Chlamydia testing should NOT be the responsibility of GPs*" and 95% disagreed with the statement "*Chlamydia testing should only be undertaken in family planning or sexual health clinic'*'. Table 1 shows that when GPs were asked to select just one preferred option, about one third believed chlamydia screening should be associated with a sexual health consultation and another third believed an organised chlamydia screening program similar to the National Cervical Screening Program with a register for recall and reminders should be introduced. Only one percent of respondents indicated that they did not think there was need for a chlamydia screening program in Australia.

**Table 1:** Preference for chlamydia screening

Statement	% (95%CI)
An opportunistic screening program capturing eligible people when they present to a GP for any reason.	25 (20, 31)
An opportunistic screening program capturing eligible people when they present to a GP for a sexual health reason (e.g.: Pap smear, contraception advice).	36 (30, 42)
An organised screening program similar to the Pap smear or breast-screening program with a system of invitation, recall and reminders.	38 (32, 44)
There is no need for a chlamydia screening program in Australia	1 (0, 3)

### Barriers and facilitators of screening for chlamydia

#### Qualitative component

GPs were asked to identify any barriers that would impact on screening for chlamydia in general practice and any facilitators that might help overcome these barriers.

##### a) Barriers

The barriers most often mentioned were GP workload, time and cost. These issues were linked to the administrative side of screening – discussing chlamydia and its possible consequences with patients and conducting follow up:

#### General practitioners are under the pump at the moment time wise, we have very little time. And that's not going to change. (DC07)

Lack of knowledge amongst GPs about chlamydia and the discomfort that some may feel in dealing with sexual health matters was also mentioned as a barrier to chlamydia screening. Some commented that GPs sometimes do not communicate well with patients and that discussing sensitive issues requires tact.

I don't think sexual history taking is taught well at medical school for a start. I think how to investigate for any sort of STIs are seen as a sort of scary. And many GPs don't have, perhaps don't even realise that a urine sample is good enough. So I think there needs to be more education targeted towards preparing GPs for this sort of work, and increasing their knowledge. And also their own self confidence. (DC13)

They don't want to umm, know about it and it takes a lot of tact if you're actually screening to not sort of dump the other partner in the poo sort of thing without them actually being guilty yet. (DC09)

GPs interviewed perceived chlamydia screening as a difficult topic to raise and discuss in general practice and worried that patients may feel insulted that they are being asked to test for a STI:

But I think the biggest thing with sexually transmitted illnesses is the problem of, if someone's supposed to be in a stable relationship they can take that as an affront you know that you suggest that maybe you should be screened for sexually transmitted illnesses. (DC09)

Patient embarrassment about STIs and patients' lack of knowledge about chlamydia were also seen as important barriers to screening. A further barrier identified was patient reluctance to respond to recalls for treatment or follow up. The administrative burden of this kind of system was felt to add to the cost and workload of a practice:

..*what do you do with people who ignore your recalls? So at the moment say for instance with our smear test, we send out two or three, I think it's, two reminders and then a third reminder comes with registered mail saying, you know, please take notice, that's the last one you're getting, rather than sending them out every month for ever, and people just don't take any notice. (DC09)*

Religious and cultural issues were also mentioned as possible barriers to screening and an area that would need to be addressed in a sensitive and culturally appropriate way. It was also suggested that some women will prefer to see a female GP for chlamydia screening.

##### b) Facilitators

Education of GPs and their practice staff was seen as important to facilitating chlamydia screening in general practice. Some GPs identified adequate financial incentives as a factor that would facilitate the implementation of chlamydia screening. A national approach to screening with national guidelines would also support screening:

..*if the health Department came out and the umm, College of Gynaecology that it was appropriate to screen women you know, every two years or every twelve months, then we would run with that recommendation.(DC07)*

Broad community education and destigmatization of chlamydia were thought to be essential prior to the introduction of broad-scale chlamydia screening:

..*you need to, I guess run a media campaign similar to a lot of the other screening media campaigns that have gone on, advising the population. And obviously you'd need to target, probably towards the younger population. (DC16)*

#### Quantitative component

##### a) Barriers

GPs considered that time constraints during the consultation was the main barrier to increased chlamydia screening. Over one third indicated that lack of a formal recall/reminder system for chlamydia screening and lack of support for contact tracing were also barriers to increasing chlamydia screening in general practice. (Table 2) Respondents did not consider that concerns about over-servicing, costs of screening to clients, difficulties in discussing sexual health issues with patients and the chance of getting a false positive test result were barriers to increased chlamydia screening in general practice.

**Table 2:** Barriers and facilitators to increasing chlamydia screening in general practice

Barrier	Probably not % (95%CI)	Not sure % (95%CI)	Probably % (95%CI)
Concerns about over-serving	74 (68, 80)	6 (3, 9)	20 (15, 25)
The cost of testing to the client	72 (70, 77)	7 (4, 10)	21 (16, 30)
Time constraints during the consultation	32 (26, 38)	6 (3, 9)	62 (56, 68)
Difficulty in talking with patients about sexual health issues	74 (68, 79)	8 (5, 12)	18 (13, 23)
The chance of getting a false positive result on testing	73 (67, 78)	16 (12, 21)	10 (7, 15)
Concerns that some pathology providers prefer swabs rather than urine specimens for chlamydia testing	67 (61, 73)	16 (12, 21)	17 (12, 22)
Patient's lack of knowledge about chlamydia	58 (51, 64)	9 (6, 13)	33 (27, 39)
Religion or ethnicity of patient	50 (43, 56)	19 (14, 24)	31 (25, 37)
Lack of a formal recall/reminder system for chlamydia testing	40 (34, 46)	19 (14, 24)	41 (35, 47)
Lack of support for partner notification/following up of the partners of positive cases	39 (33, 46)	22 (17, 28)	39 (33, 35)

Facilitators			

If there was a recognised national chlamydia screening program	4 (2, 7)	3 (1, 6)	93 (89, 96)
If payment was available for a practice nurse to discuss chlamydia testing with patients and conduct the testing	21 (16, 27)	9 (6, 13)	70 (63, 75)
If there were national guidelines recommending who should be screened and tested for chlamydia	3 (1, 6)	5 (3, 9)	92 (87, 94)
If there was an incentive payment to GPs for each chlamydia testing performed	9 (6, 13)	7 (4, 11)	83 (78, 88)
If GPs had more knowledge on how to manage chlamydia infected patients	36 (30, 43)	13 (9, 18)	50 (43, 56)
If there was an organised chlamydia education program for the general public	4 (2, 8)	5 (3, 9)	90 (85, 93)
If there was a recall/reminder system	10 (6, 14)	13 (9, 17)	77 (71, 82)

##### b) Facilitators

Over 90% of GPs surveyed indicated that they would be likely to increase chlamydia screening if there were national chlamydia screening guidelines, if there was national screening program or if there was an organised educational program for the general public. Over 80% would increase chlamydia screening if there was an incentive payment available for each chlamydia test performed. The majority of GPs consider that it is important to be able to test urines rather than swabs. (Table 2)

### Partner notification

#### Qualitative component

GPs were asked about partner notification. Over half the GPs interviewed said that they left the responsibility of partner notification up to the patient and some said that they would suggest that the partner/s should come to them for treatment or attend another clinic. Some GPs said that they prepared a letter for patients to take to partners or that they provided other documentation that could be given to partners and a third said that they had either never done any partner notification or that the situation had never arisen for them.

GPs were concerned about how to approach issues of patient confidentiality in relation to partner notification:

..*privacy and confidentiality are obviously, this whole area is one that relates to that from a personal patient's point of view. It's not something they want particularly published. So we've really got some definite areas of difficulty based on consideration of the patient's confidentiality as opposed to the community benefit. (DC06)*

They were also concerned about the sensitivity of notifying partners and the potential repercussions for relationships:

that's very touchy. If you're going to say, someone's partner has chlamydia, how do you tell them? They'll say, you didn't get it from me. It would be like saying, someone's got herpes. (DC03)

GPs also reflected on the skills required by GPs to respond to the patient if a chlamydia test is positive:

..*that's really the ramifications of performing the test. And I think this is where you talk about pre and post test counselling. So if you're going to perform the screening then you need to take some time already to prepare the patient about what would happen if there was a positive result. (DC13)*

GPs saw assistance with partner notification as a priority if a chlamydia screening program were to be introduced. This service should be presented in such a way so as to minimise any perceived stigma that may be attached to a STI:

..*especially if it happens automatically where it's you know, it happens that they do it as a routine. So people don't feel like they're being singled out as you know, it's a public issue so it's reasonable to trace any infectious disease, whether it's sexual or not.(DC09)*

#### Quantitative component

Again we found similar results using quantitative methods. GPs tend to rely on patient referral with the vast majority indicating that they ask the patient to follow up their contacts; about one quarter indicated that they gave their patient a contact letter to give to their partners. A small proportion indicated that they did nothing because they assumed that health department undertook partner notification when a new case of chlamydia was notified.

When asked what support or resources GPs would like to help them with partner notification, over two thirds indicated that contact letters would be helpful and about half specified that websites or the officers employed by the health department to assist with contact tracing would be helpful. Interestingly, only a small proportion (7%) indicated that SMS phone messages would be helpful. (Table 3)

**Table 3:** Partner notification practices and resources

Partner notification practices	Proportion* % (95%CI)
Ask the patient to follow up their contacts	92 (88, 95)
Give the patient a contact letter to give to their contacts	23 (18, 292)
Write a letter to the patient's contacts directly	2 (0.4, 4)
Directly ask the health department partner notification officers to follow up their contacts	14 (10, 19)
Nothing – the health department does the partner notification whenever a new case of chlamydia is notified	8 (5, 12)

Partner notification resources	

Partner/contact letters	70 (64, 75)
Websites	42 (36, 48)
SMS phone messages	7 (4, 10)
Telephone hotline	31 (25, 37)
Health department partner notification officers	50 (44, 56)

* Multiple responses possible

## Discussion

Our study is the first detailed qualitative study of chlamydia screening in Australia and has identified a number of issues that are important to address before chlamydia screening is introduced widely within the Australian health care setting. Firstly, GPs felt strongly that screening would be easier if there was a national screening program and if the public had a greater understanding of chlamydia. Secondly, they felt that incentive payments would facilitate screening. Thirdly, greater support for contact tracing would be important if screening is to increase. These findings are completely in line with the views of a recent study of Australian women we conducted that found that women want chlamydia screening to be 'normalised' – to become part of a routine health check that is based on their current age, not their sexual history [21].

Most GPs interviewed believed that chlamydia screening should ideally be opportunistic and offered during a sexual health consultation. While linking chlamydia screening with a Pap test may make it easier for GPs to introduce the subject of chlamydia with their patients [22], cervical screening in Australia currently commences at age 20 and as a result, other resources would be needed to ensure women under 20 years of age are also screened. Further, it is likely that the introduction of the human papilloma virus (HPV) vaccination will lead to revisions in the National Cervical Screening Program in Australia with the commencement of screening being delayed to a later age [23].

GPs completing the postal survey were more likely to believe that an organised screening program with a system of recall and reminders would be the preferred model for screening in Australia rather than relying on opportunistic screening alone. The reasons for this discrepancy between the qualitative and quantitative results are unclear, but differences in methodology (quantitatively with a closed answer response versus a qualitative interview question), may explain the difference. Nevertheless, their support for chlamydia screening in Australia was encouraging. However, given that Australian GPs are currently screening only about 8% of women under 25 years of age each year [24], this suggests that although they support screening, there must be considerable barriers to increased screening. Mathematical modelling of chlamydia screening shows that 20–30% of women must be screened each year to have an impact on lowering chlamydia prevalence [25,26] highlighting that any chlamydia screening program must increase the proportion screened each year.

We found that GPs identified a number of important barriers that must be addressed for screening to be successful. Time was seen as the greatest barrier to increased chlamydia screening. Patient embarrassment and lack of knowledge about chlamydia were also mentioned as important barriers to screening. This finding combined with our observation above that many GPs would prefer to screen for chlamydia in the context of a sexual health consultation, raises the possibility of prompting that all women from age 16 have a regular women's health check that could for example, include chlamydia and STI screening in the younger age groups and Pap smear screening as the women move into their mid 20s. This would normalise chlamydia screening, by placing it in the context of a woman's regular health maintenance. This approach has previously been found to increase acceptability of chlamydia screening amongst young women [15]. Surveys of GPs conducted both overseas [27-29] and in Australia [30] have also found that lack of time and fear of embarrassing patients are commonly expressed barriers to increased chlamydia screening by general practitioners.

GPs suggested several different facilitators for increased screening including financial incentives for GPs; funding for practice nurses; education for GPs, practice nurses and the general public; national screening guidelines; and the introduction of a formal screening program with associated recall mechanisms. In view of the low screening rates in Australia, it is vitally important that these different facilitators are investigated when designing Australia's screening program [31]. Since November 2006, practice nurses in Australia have been able to conduct chlamydia testing if it is undertaken concurrently with a cervical smear (Pap test), but they are unable to conduct screening otherwise. However, only about 55% of clinics in Australia include a practice nurse [32] and as practice nurses play an important role in the National Chlamydia Screening Program in the United Kingdom, further consideration should be given to expanding their role in Australian general practice [33].

Partner notification is an essential component in the control of STIs [34]. Our participants indicated they generally leave the responsibility of partner notification to the patient, with about one quarter indicating that they prepared a letter or documentation for patients to take to their partners. These findings are consistent with a recent postal survey of Australian GPs which also found that the majority of GPs believed that patients were largely responsible for contacting partners [35].

Our study has a number of strengths. Firstly, it is one of the few studies of GPs' attitudes to chlamydia screening that has included both a qualitative and quantitative component. The findings of the postal survey were generally in concordance with those obtained in the qualitative interviews. We obtained a response rate of 47% for the postal survey, a reasonable response for a postal survey of GPs, particularly as reported response rates have dropped over the last two decades [36] and a recent postal survey of GPs in New South Wales reported a response rate of 45% [37].

There are some limitations in our study results. Firstly, the GPs selected for the qualitative interviews may represent a biased sample; with a response rate of 30% it is possible that those interested in STIs were more likely to participate in the study. Secondly, female GPs and those aged 45 to 54 years were over-represented in the postal survey. As previous surveys have shown that female GPs were more likely to test asymptomatic patients and had a superior knowledge about appropriate specimens for diagnosing Chlamydia [38], it is possible that the postal survey results may have some bias and led to an overestimation of GPs' support for chlamydia screening. Thirdly, our sample size of 255 was lower than the 300 calculated, reducing the precision of our estimates slightly. Finally, this study was conducted in Victoria and may not be generalizable to rest of Australia. However, with the exception of remote areas, the demographic profile of GPs is similar across the different States of Australia [20] and the prevalence of chlamydia among non-Indigenous populations across Australia is also similar [3,39].

## Conclusion

Australian GPs believe that general practice is an appropriate place for chlamydia screening to take place, particularly in the context of a sexual health consultation where chlamydia could be normalised. However, if screening is to succeed in Australia and screening rates are to increase to the levels necessary to have an impact on the burden of chlamydia, policy makers must investigate the facilitators suggested here such as incentive payments, funding for practice nurses, education campaigns and recall/reminder mechanisms. These relatively straight forward changes have the potential to substantially increase chlamydia screening.

## Competing interests

The authors declare that they have no competing interests.

## Authors' contributions

JH contributed to study design, supervised administration of the postal survey and drafted the manuscript. RP and NP contributed to study design, conducted all interviews, analysed the results and contributed to the manuscript. JG contributed to study design, provided advice regarding the interviews and contributed to the manuscript. CF conceived the study, contributed to study design and the manuscript.

## Pre-publication history

The pre-publication history for this paper can be accessed here:


